# Mental Illness in the Post-pandemic World: Digital Psychiatry and the Future

**DOI:** 10.3389/fpsyg.2021.567426

**Published:** 2021-04-16

**Authors:** Muhammad Omair Husain, David Gratzer, Muhammad Ishrat Husain, Farooq Naeem

**Affiliations:** ^1^Department of Psychiatry, University of Toronto, Toronto, ON, Canada; ^2^Centre for Addiction and Mental Health, Toronto, ON, Canada

**Keywords:** telepsychiatry, digital health, COVID-19, digital psychiatry, mental health

## Background

The World Health Organization declared the novel coronavirus disease 2019 (COVID-19) outbreak a global health emergency in January 2020. Rates of infection and consequently, mortality have risen rapidly, resulting in a global pandemic. With no evidence-based treatments available, most countries have implemented quarantine measures to mitigate the spread of the virus. The world has largely focused on the physical suffering associated with COVID-19. However, the mental health sequelae of the pandemic are beginning to gain deserved attention. COVID-19 poses unique challenges to population mental health, given the colossal societal impact of nationwide lockdowns and health services struggling to cope. Mental health and well-being have been adversely affected by direct exposure to the virus (e.g., depression, anxiety, grief, suicidality) and from the social and economic upheaval that is occurring at an individual and population level (Reger et al., 2020; Wind et al., 2020). The economic down turn in the context of COVID-19 is a major concern, with psychological distress and suicide rates potentially rising on the back of sustained financial stress (Reger et al., 2020). The Severe Acute Respiratory Syndrome (SARS) epidemic of 2003 has taught us that psychological distress can affect both patients and clinicians treating them during an outbreak and continue in its aftermath (Lee et al., 2007). Amidst COVID-19, health services have had to adapt rapidly, implementing new ways of working to meet the rising demands of the population. However, across health settings, there is considerable variation with some institutions lacking the necessary resources, infrastructure, training, and support to allow clinicians to deliver mental health care in an era of physical distancing. Based on collective clinical experience, we provide a commentary on the rapid transition from in-person or traditional psychiatric care, to virtual mental health care (telepsychiatry) in response to COVID-19 and discuss the advantages, disadvantages and implications of digital psychiatry now and in the post-pandemic world.

## COVID-19 and the Acceleration of Digital Psychiatry

Over the past two decades, digital psychiatry has continued to evolve as a cost-effective means to improve access to care for psychiatric patients. Digital psychiatry is an umbrella term that includes digital interventions delivered through applictions (web and mobile based) as well as the delivery of telemental health care through virtual (videoconferencing) platforms. The majority of this commentary focuses on the latter, which we will refer to as telepsychiatry and virtual care. The burden of mental disorders has not been matched by the appropriate resources and services necessary to treat them, resulting in a large mental health treatment gap. There is hope that digital psychiatry can bridge this gap, improving access to treatment, and hence outcomes for people with mental disorders. Evidence supports the use of digital psychiatry as a feasible platform, acceptable to users as well as effective in improving outcomes and quality of life across a variety of mental disorders (Bashshur et al., 2016). Literature is primarily based on mood and anxiety disorders, with an under-representation of psychosis (Bashshur et al., 2016). Furthermore, the intervention modality most commonly used in digital mental health studies is Cognitive Behavior Therapy (CBT) (Bashshur et al., 2016). Therefore, caution must be exercised when generalizing the enthusiasm of digital mental health care across the spectrum of severe mental illness. Nonetheless, there are published best practice guidelines on the use of virtual platforms for the delivery of care by the American Telemedicine Association and the American Psychiatric Association (Shore et al., 2018). In Ontario, Canada, the Ontario Telemedicine Network (OTN) a non-profit initiative was formed in the late 1990's as a means to connect individuals in remote areas to quality care (Brown, 2013). Telepsychiatry has continued to develop over the past two decades with evidence supporting it's equivalence to face-face care with regards to therapeutic alliance and patient satisfaction (Hilty et al., 2013; Parish et al., 2017). Although there are few randomized controlled trials comparing traditional in-person mental health care with virtual care, evidence from systematic reviews and meta-analyses have indicated that treatment effects are largely equivalent when both approaches are compared (Bashshur et al., 2016; Langarizadeh et al., 2017; Batastini et al., 2020).

Although telepsychiatry has faced a number of obstacles to implementation, in the context of the COVID-19 pandemic, many perceived barriers to the adoption of virtual care have now been overcome across the world (Mann et al., 2020; Perez Sust et al., 2020). The meteoric rise of telepsychiatry has been felt world over. In the UK, the National Health Service facilitated the rapid ramp up of virtual psychiatric care despite pre-existing cautionary guidance from the Royal College of Psychiatrists (Dave et al., 2020). The Australian health service provider, Medicare, moved to support telehealth solutions to allow mental health services to provide care seamlessly despite physical distance restrictions being placed on the population (Davenport et al., 2020). In the 6-weeks between mid-March and April, 2020 when Medicare began funding telemental health, 35 million Australian dollars were spent on these services (Rosenberg et al., 2020). Countries like Israel, Singapore, Korea and Taiwan have successfully managed the COVID-19 pandemic by responding rapidly and relying heavily on technology (Salvador-Carulla et al., 2020). Although low and middle-income countries have seen a rise in telepsychiatry as well, the full potential of using virtual platforms in these settings is yet to be realized (Naeem et al., 2020). Telepsychiatry represents a viable resource in overcoming the significant mental health gap in low and middle-income countries. However, the proliferation of telepsychiatry in these contexts is often undermined by lack of telehealth infrastructure, national telehealth policies, data governance frameworks and lack of training and education on the use telehealth technologies for the health-workforce (Naeem et al., 2020).

## Virtual Care at the Centre for Addiction and Mental Health (CAMH)

The COVID-19 pandemic poses a serious challenge for mental health services across the globe. There has been a rapid uptake of virtual healthcare delivery by a number of mental health providers in preparation for the mental health pandemic that is likely to follow COVID-19. Pre-pandemic, the Centre for Addiction and Mental Health (CAMH), Canada's largest mental health teaching hospital, prioritized the innovation and expansion of telepsychiatry services. However, the implementation of these services across the organization had taken years to fully establish. Since the start of the COVID-19 pandemic, the acceleration of telepsychiatry has been meteoric, with virtual platforms being utilized to deliver patient care. In the US this uptake of virtual care has been facilitated by the relaxation of privacy legislation, augmentation of physician remuneration policies, and prescribing policies (Torous et al., 2020). Similarly, in Canada there has been a change in regulations and the introduction of COVID-19 specific billing codes as a means to increase access to virtual care across medical specialties (Ontario Ministry of Health Ministry of Long-Term Care, 2020). CAMH became entirely digitized in a matter of days, successfully expanding virtual care provision to meet the mental health demands of the population. In 2019 CAMH delivered virtual care to over 3,000 patients from over 550 communities across Ontario (D'Andrea, 2020). This figure increased by over 750 percent from March to April of 2020, with over 3,000 virtual care visits being provided per month (D'Andrea, 2020). The number of mental health providers trained to deliver virtual care also increased from 30 practitioners to over 400 (D'Andrea, 2020). CAMH is an example of the adaptability mental health services need to display in order to meet the demands of the populations they serve. In our experience, feedback from colleagues and patients has been overwhelmingly positive. Clinical staff are finding that remote working has provided more flexibility in their clinical schedules as well as making them more time-efficient. Feedback from service users echo the flexibility and convenience aspect, but also report cost and time saving (less waiting, no cost of transport or parking). Dr. Catherine Zahn, President and CEO of CAMH, has stated that “*virtual health platforms will be a permanent and growing fixture of the healthcare system, and be offered as an accessible, flexible and secure mental health care option for patient care going forward.”* CAMH is now creating training programs for other hospitals and community-based healthcare providers to help scale capacity to deliver mental health services virtually elsewhere.

## Potential Pitfalls of Digital Psychiatry

Despite its advantages, telepsychiatry is not a panacea, and there are potential harms that must be considered. Virtual care is inherently associated with a degree of risk where data breaches and compromise to patient confidentiality and privacy are concerned (Lustgarten et al., 2020). The use of virtual platforms with built-in data encryption, adware, and malware protection, as well as firewalls, has been endorsed as a means to mitigate risks of data breaches (Barnett, 2019). Furthermore, ensuring robust consent procedures is of utmost importance. During this process, the risks to privacy and confidentiality should be clearly explained to patients, along with the limitations of the safeguards that are in place to prevent breaches. Patients should be advised to engage in consultations in a private space as confidential information may be discernable to individuals in their proximity. When delivering telepsychiatric care there are legal implications related to jurisdiction and licensure, where provision of care must be restricted to the province or state that the practitioner is licensed in. Confirmation of identity and identity fraud is another legal concern. There are also implications with regards to involuntary admissions and collaborative arrangements with law enforcement, health and social services to facilitate them. Ultimately, there is the inevitable risk of being unable to respond to psychiatric emergencies in a timely manner. All virtual health platforms inherently carry this risk and therefore guidance from the APA and the OTN pertain specifically to it, with advice that clinicians should ensure there is consistency in where the patient is located during sessions, along with having access to emergency contact information (a friend or family member of the patient) and knowledge of local resources in the event of a crisis (e.g., police, emergency medical services etc.) (Shore et al., 2018).

It is important to recognize that though virtual platforms can improve access to care for some individuals, others may not be able to take advantage of them (Strudwick et al., 2020). There are many barriers to successful uptake of telepsychiatry services including accessibility (e.g., access to internet and smartphones), user ability, provider competency, as well as language and cultural appropriateness (Strudwick et al., 2020). Patients with schizophrenia are known to have cognitive impairment (Husain et al., 2018), which may hinder their ability to engage with telepsychiatry. People experiencing homelessness may also be disproportionally excluded from accessing appropriate care and interventions if largely delivered virtually. Individuals in later life may be less proficient with virtual platforms. To address these factors, a collaborative approach with all relevant stakeholders (service users, clinicians, academics, policymakers) is necessary to develop telepsychiatry services that are inclusive and meet the diverse needs of the population. We must be cautious not to widen the existing mental health gap for already hard-to-reach groups, including people from racialized communities and those living in rural areas where connectivity may be limited. Hard-to-reach individuals are often those with the highest need for care and it is essential that future developments in telepsychiatry ensure parity of access.

We acknowledge that telepsychiatry is not a “one size fits all.” There may well be certain patient groups that are not suitable for virtual care but the literature on the unintended harms of telepsychiatry is scant. Although telepsychiatry has been reported to be effective and satisfactory to addiction medicine patients, there are no reports to our knowledge on the use of virtual platforms for individuals with internet or gaming addiction. There is a need to develop the evidence base for potential unintended harms associated with virtual care in the future. How much is too much? The potential impact of screen time on cognitive function and executive function is yet to be determined (Minielly et al., 2020). Nonetheless, in the context of the ongoing global pandemic at present the balance remains in favor of telepsychiatry, vs. no care at all.

## Digital Psychiatry in the Post-Pandemic Setting

Although the rapid uptake of virtual care has been a product of necessity, the COVID-19 pandemic may have fundamentally changed the way mental health care is delivered. Historically, reluctance to accept telepsychiatry has been centered around the building of rapport and therapeutic relationships (Torous and Wykes, 2020). Appropriate investment is needed for clinician training to deliver virtual care, which has the potential to help the many who will experience mental health fallout from the COVID-19 pandemic but may also redefine “*digital mental health as simply mental health*” in the post-pandemic era (Torous et al., 2020). Furthermore, investment is required in the development of new technologies alongside the development and testing of apps to support the delivery of digital health. We must consider the enhancement and repurposing of existing infrastructure, for example phonebooths equipped with built-in tablet devices that would allow individuals without access to digital platforms to engage. Parity of care could also be achieved by initiatives that offer mobile devices to those unable to afford them. “Virtual walk-in centres” can also be developed by allocating spaces in hospitals and walk-in centres where patients can utilize designated computers or tablet devices. Digital health literacy needs to be enhanced through targeted education interventions to reduce the disparity in access to digital care. These strategies would enhance digital inclusion and potentially address health inequality in the future where digital platforms are likely to form a large part of healthcare delivery (Farooq et al., 2015). The evolution of digital health care requires clinicians, academics, funders, developers and policy makers to collaborate and develop the evidence base of real-world pragmatic effectiveness trials of virtual care platforms, e-therapies, apps, wearable technology and actigrapy. Future directions for research include rigourous and robust clinical effectiveness trials that compare virtual care with in-person care, as well as clinical trials including a variety of mental health presentations and service settings. We will only then be able to determine what works best for who. More research is also needed on the use of mobile applications and artificial intelligence (AI). AI informed mobile applications have the potential to offer personalized real time intervention and provide immediate response to the changing needs of the patient. We have achieved Digital Psychiatry 1.0, allowing us to connect better with our patients (virtual care vs. in person). Digital Psychiatry 2.0 needs to move past connection and toward the empowerment of patients through remote monitoring of their mental health and real-time interventions by clinicians. Such a platform would not only improve access to mental health care, but invariably lead to a more responsive mental health care system that would improve patient outcomes in the post-pandemic world.

## Author Contributions

MOH and MIH conceived the idea for this paper and led in the writing of the manuscript. DG and FN helped with drafting the manuscript. All authors approved the final draft prior to submission.

## Conflict of Interest

The authors declare that the research was conducted in the absence of any commercial or financial relationships that could be construed as a potential conflict of interest.
